# Antibiotic resistance genes are more abundant in microplastic textile biofilms than natural cotton biofilms in freshwater

**DOI:** 10.1128/aem.00719-26

**Published:** 2026-07-10

**Authors:** Nhung H. A. Nguyen, Anna Bohmova, Jana Novotna, Jakub Wiener, Jakub Riha, Tham C. Hoang, Alena Sevcu

**Affiliations:** 1 Institute for Nanomaterials, Advanced Technologies, and Innovation, Technical University of Liberec, Liberec, Czechia; 2 Department of Material Engineering, Faculty of Textile Engineering, Technical University of Liberec 48261, Liberec, Czechia; 3 School of Fisheries, Aquaculture, and Aquatic Sciences, College of Agriculture, Auburn University, Auburn, Alabama, USA; 4 Faculty of Science, Humanities and Education, Technical University of Liberec, Liberec, Czechia; University of Minnesota Twin Cities, St. Paul, Minnesota, USA

**Keywords:** antibiotic resistance genes, nylon, polyester, acrylonitrile, cotton, freshwater, microplastic fibers, biofilms

## Abstract

Microplastic fibers (MPFs) are widespread pollutants in freshwater systems, providing artificial surfaces that facilitate microbial attachment and the potential spread of antibiotic resistance genes (ARGs). We compared bacterial colonization on natural cotton fibers with that on synthetic MPFs (Kevlar, acrylonitrile, polyester, and nylon) incubated in river and lake water. Bacterial biomass and community composition were analyzed using epifluorescence microscopy, scanning electron microscopy, and 16S rRNA sequencing, while the presence and relative abundance of key ARGs (*bla*NDM-1, *bla*KPC, and *bla*OXA-48) were quantified using qPCR. Cotton fibers developed substantially higher biofilm loads than any synthetic MPF, supporting dense and taxonomically diverse microbial communities. In contrast, synthetic MPFs supported lower levels of bacterial colonization but exhibited significantly higher levels of ARG enrichment, with *bla*OXA-48 showing the highest relative abundance. Several taxa, including *Fluviicola, Sphingobium, Nitrospira, Schlesneria*, and TRA3-20 (*Burkholderiaceae*), harbored ARGs across all synthetic MPF types. Overall, the findings highlight a clear difference in biofilm quantity and ARG prevalence, with cotton accumulating the most biofilm but having the lowest ARG burden, whereas synthetic MPFs supported ARG-associated bacteria despite lower colonization. These results suggest that synthetic MPFs may play a disproportionately large role in the environmental dissemination of antibiotic resistance due to their mobility and affinity for ARG-harboring microbial communities in freshwater ecosystems.

**IMPORTANCE:**

Microplastic fibers (MPFs) are widespread in freshwater systems but remain underexplored as reservoirs and vectors of antibiotic resistance. This study reveals that synthetic MPFs serve as enriched niches for bacteria harboring relevant antibiotic resistance genes (ARGs), in contrast to natural fibers like cotton. By combining high-resolution microscopy, 16S rRNA gene sequencing, and quantitative PCR, we demonstrate that MPFs selectively support ARG-bearing taxa, including Fluviicola and Sphingobium, across multiple fiber types. These findings suggest that MPFs in aquatic environments may facilitate horizontal gene transfer and contribute to the environmental dissemination of antibiotic resistance. Understanding microbial colonization patterns on MPFs is critical for assessing the ecological and public health risks posed by microplastic pollution.

## INTRODUCTION

Microplastic fibers (MPFs), defined as thin plastic fibers < 5 mm in length, are a significant contributor to microplastic pollution in aquatic environments (1, 2). The primary sources of MPFs are industrial activities, such as the manufacturing of synthetic fiber materials, and laundering clothes made from these textiles (3). It has been reported that the laundering of a single garment can release over 1,900 fibers most of which pass through wastewater treatment plants and enter freshwater and marine ecosystems (4–7).

Microfibers from natural textiles, such as cotton, are also abundant in aquatic environments, and these originate primarily from textile production, household washing, and release of fibers from discarded clothing (8). Natural fibers are composed of cellulose and have a hydrophilic surface, making them more readily biodegradable. In contrast, synthetic MPFs are typically hydrophobic polymers, such as polyester, nylon, or acrylonitrile, and degrade more slowly. Both types of fibers serve as substrates for bacterial colonization and biofilm formation, thereby influencing microbial community structure and dynamics. Notably, bacterial communities attached to these fibers may harbor antibiotic resistance genes (ARGs) that can spread through horizontal gene transfer, presenting ecological and public health concerns (9, 10).

Although the effects of MPF pollution on aquatic organisms have been studied, the interactions between microbial communities and MPFs in freshwater ecosystems are not well understood, particularly compared to natural fibers. Biofilms act as external digestive systems by trapping enzymes in extracellular polymeric substances and concentrating them on substrate surfaces, such as microplastics, thereby enhancing biodegradation efficiency (11–13). Conversely, biofilms can serve as reservoirs for ARGs, providing a protected environment that facilitates gene exchange and shields bacteria from antimicrobial stress (10, 14). Bacteria such as *Pseudomonas*, *Bacillus*, and *Flavobacterium* colonize both synthetic and natural fibers, influencing their persistence and ecological impact (15–20). Owing to their cellulose content, cotton fibers support a broader range of cellulolytic bacteria, such as *Cellulomonas* and *Streptomyces*, which enhance natural degradation processes (21–23). In contrast, synthetic fibers degrade slowly, allowing for prolonged microbial colonization and accumulation of environmental pollutants, including antibiotics (24, 25). Therefore, comparisons between synthetic and natural fibers are crucial to understanding both biofilm-mediated ARG dissemination and fiber biodegradation in freshwater systems.

The Lužická Nisa River is a transboundary waterway that flows through the Czech Republic, Germany, and Poland. It receives a mixture of urban, industrial, and agricultural inputs, including wastewater rich in microplastics, antibiotics, and antibiotic-resistant bacteria. These conditions create an ideal environment for biofilm formation on microfibers and the potential exchange of ARGs. Studying this river will shed light on microbial colonization dynamics and the environmental risks associated with natural and synthetic fibers (26).

This study assessed the following: (i) bacterial communities attached to a common environmentally relevant natural fiber (cotton) and synthetic MPFs from textile materials (Kevlar, acrylonitrile, polyester, and nylon); (ii) the occurrence of ARGs within these biofilm communities; and (iii) the physical characteristics of MPFs and potential associated microorganisms. By integrating microbial and material analyses, this work provides a comprehensive understanding of microfiber-associated biofilms in freshwater systems and their implications for the dynamics of ARGs in river water.

## MATERIALS AND METHODS

### Microplastic fiber preparation and characterization

Natural cotton fibers, sourced from untreated 100% cotton textiles, were used to ensure environmental relevance, while synthetic fibers (para-aramid [Kevlar], acrylonitrile, polyester, and polyamide 6-6 [nylon]) were sourced from the commercial supplier Goonvean Fibers Ltd (UK). The provider reported the following fiber densities: 1.54 g/cm^3^ for cotton, 1.44 g/cm^3^ for Kevlar, 1.35 g/cm^3^ for nylon, 1.17 g/cm^3^ for acrylonitrile, and 1.38 g/cm^3^ for polyester. Prior to testing, the fibers were washed (100 g per 2 L) with an anionic tenside surfactant solution (1 g/L) for 30 min at 60°C to remove chemical lubricants from the manufacturing process, after which the fibers were filtered and dried at 60°C in a laboratory oven.

Surface images of the fibers were acquired using a VEGA 3 TESCAN scanning electron microscope (SEM; TESCAN GROUP a.s., Czech Republic), operated with an accelerating voltage of 5 kV at low probe current (ca. 15 pA). Fiber diameter measurements were made using a 20 µm In-Lens secondary electron detector with SmartSEM software and associated imaging software (27).

### Microplastic fiber length and width distribution

The size and distribution of MPFs were determined using methods described by Barrick et al. (27). Briefly, 0.1 mg of each MPF was mixed with 10 mL of 70% ethanol to create a homogeneous microfiber suspension. Then, three 0.1 mL replicates were filtered onto a 1.0 µm Sartorius filter fitted with a grid. Individual microfibers were counted, and the length and width of each fiber were recorded using an upright Nikon Eclipse C*i*-L Plus microscope (Nikon Instruments Inc., USA) fitted with a Ds-F*i* 3 camera and NIS-Elements imaging software.

The length and width measurements were used to assess microfiber size distribution based on a cumulative frequency distribution. This method is often used to assess toxicant exposure and effect distribution for ecological risk assessments (28, 29). Additionally, cumulative log-logistic distributions were prepared for each microfiber type using least squares regression. The data were ranked from shortest to longest, and the centile calculated for each microfiber using equation 1:


(1) $$Centile=Rank\;\times \;\frac{100}{\left(n\;+1\right)}$$

where *n* is the total number of microfibers. Finally, the 10th, 50th, and 90th cumulative percentiles of microfiber length and width were calculated, with the 10th and 90th percentiles representing the size range of 80% of microfibers in this study. Previously, these percentiles were used to represent effect and exposure benchmarks, respectively, in ecological risk assessments.

Based on the assumption that the fibers have a cylindrical tube-like shape, microfiber surface area was calculated by summing the areas of the two circular ends of each fiber and the curved surface area, using equation 2:


(2) $$Area\;=3.1416\times \left(\left(d\times l\right)+\frac{d^{2}}{2}\;\right)$$

where *d* and *l* are the diameter and length of the fiber, respectively.

### Surface roughness

Six linear roughness characteristics were selected to evaluate the surface roughness of cotton, Kevlar, acrylonitrile, polyester, and nylon MPFs, that is, Rp (maximum peak height), Rv (maximum valley depth), Rz (maximum height), Rc (mean height), Rt (maximum profile height), and Ra (arithmetic mean height). All roughness measurements, along with mean height (Ra), maximum height (Rz), and maximum profile height (Rt), were performed under a Lext OLS 3000 IR confocal microscope with associated image analysis software (Olympus America Inc., USA).

### Experimental design and water collection

The river water used in the experiments was collected from the Lužická Nisa River in the town center of Liberec, Czech Republic (50°45'59.846"N, 15°3'8.993"E). The water was transported to the microbiology laboratory in a clean 20 L plastic container, and the experiments began immediately upon arrival. First, the river water was passed through a 20 µm pore Whatman filter (Sigma-Aldrich, US), after which a suspension of each MPF type was prepared in triplicate in a sterilized 1 L glass bottle at a concentration of 100 mg/L. The suspension was thoroughly mixed at the beginning and then left without agitation. Control samples with no MPFs were also prepared simultaneously. All bottles were then incubated in the dark (to prevent algal growth) at laboratory temperature (20 °C) for 4 weeks. Subsamples of 150 mL were taken from each bottle after 1 week and 4 weeks for bacterial community analysis.

### Microscopy analysis of microplastic fiber colonization

Bacterial attachment on MPFs was assessed after 1 week and 1 month. Briefly, the samples were stained using the Live/Dead BacLight Staining Kit (Life Technologies, USA) and then examined under an Axio Imager epifluorescence microscope (Zeiss, Germany). Images of bacterial colonization on each MPF were produced using a Zeiss ULTRA Plus SEM (Zeiss Group, Germany). For more detailed information on the procedures used, see the study by Nguyen et al (30).

### DNA extraction

After 1 week and 1 month, a 150 mL aliquot of each sample was collected and passed through a 20 μm Whatman filter (Sigma-Aldrich, US) to capture any attached bacteria, and then through a 0.2 µm filter (Sigma-Aldrich, US) to capture any free-living bacteria. DNA was extracted from both filters using the FastDNA Spin Kit for Soil (MP Biomedicals, USA), according to the manufacturer’s protocol. The DNA yield was then determined using a Qubit 2.0 Fluorometer (Life Technologies, USA). For more detailed information on the procedures used, see Nguyen et al. (20).

### Detection of antibiotic resistance genes using qPCR

To detect ARGs in microfiber-associated bacterial communities, quantitative PCR (qPCR) assays were performed using a LightCycler 480 system (Roche, Switzerland) under the reaction conditions described in our previous study (31). Three clinically relevant *β*-lactamase genes were analyzed (*bla*NDM-1, *bla*OXA*-*48, and *bla*KPC); these ARG subtypes were selected based on their prevalence in wastewater and freshwater environments, their clinical importance (due to conferring resistance to broad-spectrum *β*-lactam antibiotics), and their prior association with microplastic biofilms in the Czech Republic (32).

For relative quantification, the cycle threshold (Ct) values were converted to relative abundance using the delta Ct method, with cotton fibers serving as the reference sample. Lower cycle quantification (Cq) values indicate higher amounts of the target sequence, while higher Cq values indicate lower amounts (33). Briefly, relative abundance (Eff) can be described using equation 3:


(3) Eff =(Ct sample−Ct ref)

Since the relative quantity of the reference sample (cotton) is 1, a sample relative abundance of 2 indicates twice the amount of target DNA in the sample compared to the reference sample (31).

### Microbial community profiling

The V4 region of the 16S rRNA gene was amplified using primers 515 F (5′–GTGYCAGCMGCCGCGGTAA–3’) and 806 R (5’–GGACTACNVGRGTWTCTAAT–3’), following the method described by Caporaso et al. (34), with slight modifications. In brief, the temperature sequence for PCR cycle conditions during the first PCR was as follows: 95°C for 3 min; 10 cycles at 98°C for 20 s, 50°C for 15 s, and 72°C for 45 s, followed by a final extension at 72°C for 1 min. For the second PCR, which used fusion primers and the first PCR product as a template, the conditions were as follows: 95°C for 3 min; 35 cycles at 98°C for 20 s, 50°C for 15 s, and 72°C for 45 s; and a final extension at 72°C for 1 min. Finally, the concentration of purified PCR product was measured using a Qubit 2.0 fluorometer (Thermo Fisher Scientific, USA). Barcode-tagged amplicons from the different samples were then mixed in equimolar concentrations. Sequencing of bacterial amplicons was performed on the Genexus IonTorrent platform (Thermo Fisher Scientific, USA). For more detailed information on the procedures performed, see a study by Němeček et al. (31).

The data thus obtained were processed using QIIME 2 2023.2 software (35). First, the raw sequence data were demultiplexed and quality-filtered using the q2-demux plugin, after which denoising was performed using DADA2, via the q2-dada2 plugin (36). First, taxonomy was assigned to the amplicon sequence variants (ASVs) using the QIIME 2-feature-classifier (37). Next, the ASVs were classified using classify-sklearn naive Bayes against the SILVA 138 database (38), after which mitochondria and chloroplast data were removed from the data set. Accuracy of classification was evaluated against a D6306 ZymoBIOMICS Microbial Community DNA Standard (Zymo Research, USA).

### Data analysis

The QIIME 2 outputs were processed using the phyloseq (39) and vegan (40) packages in the R software environment. Alpha diversity metrics (Observed, Shannon, Simpson, and Inverse Simpson) were computed on rarefied data (subsampled without replacement). Principal coordinates analysis (PCoA) was used to compare bacterial communities across samples, and Bray-Curtis dissimilarity was used to measure differences between communities based on their relative abundances without rarefaction. Additionally, taxonomy bubble plots were created using the same relative abundances but including taxonomic bins with a mean relative abundance >0.005 only (ASVs aggregated at a given level). A permutational MANOVA test (adonis2 function in the vegan package) was performed to determine the impact of fiber material on bacterial composition, with pairwise.adonis (41) applied to test for significant effects. Finally, Spearman correlations were used to determine the strength and direction of the relationship between selected qPCR markers and the relative abundance of the dominant taxonomic bins.

## RESULTS

### Microplastic fiber size distribution

**Fig 1:**
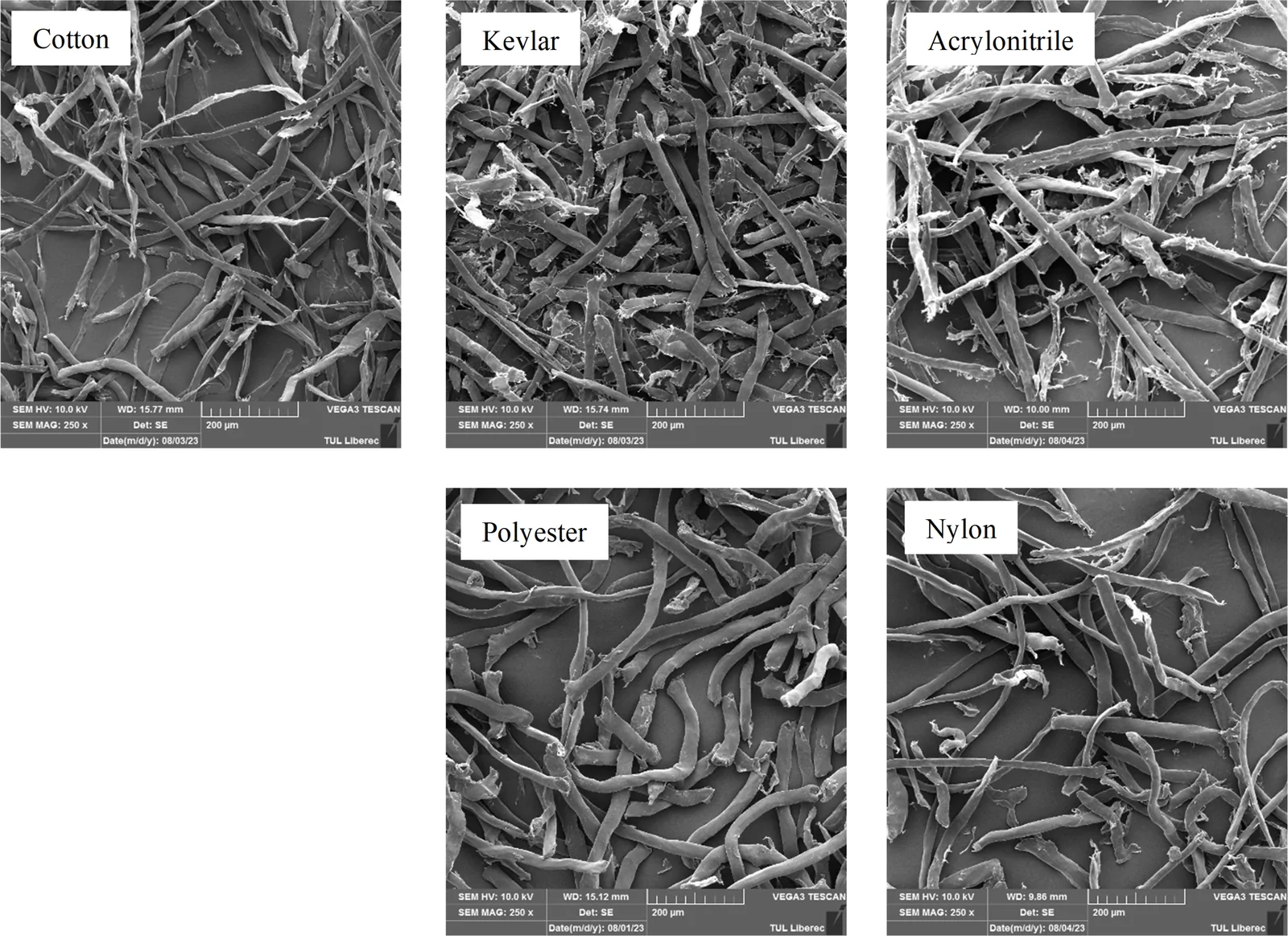
MPF morphology under SEM with a scale bar of 200 µm.

SEM images revealed that the ends of Kevlar and acrylonitrile fibers were frayed, whereas the ends of cotton, polyester, and nylon fibers appeared much smoother (Fig. 1).

Minimum and maximum lengths and surface areas tended to increase in the order cotton > Kevlar > polyester > acrylonitrile > nylon, although the maximum length tended to be greater in Kevlar than in cotton, and the maximum width was higher in acrylonitrile than in nylon (Table 1). Nylon fibers tended to be longest, with 80% of fibers between 770 and 5,400 µm, followed by acrylonitrile fibers with 80% between 303 and 830 µm. Kevlar fibers tended to be shortest, with 80% between 195 and 576 µm. Fiber diameter differed only slightly between fiber types, with polyester fibers being the widest and cotton fibers narrowest. Overall, nylon fibers had the largest average surface area (369,331 µm^2^), along with greatest variation in area, while cotton fibers had the smallest average surface area (24,729 µm^2^) and lowest variation (Table 1).

**TABLE 1:** Range and percentile of cumulative frequency for cotton and synthetic microplastic fiber length, width, and mean surface area

Fiber type	Length (µm)				Width (µm)				Area (µm^2^)
	Range	50th	10th	90th	Range	50th	10th	90th	
Cotton	91–811	368	202	597	9–36	20	14	28	24,729 ± 12,019
Kevlar	147–762	345	195	576	12–42	25	17	33	29,142 ± 12,732
Polyester	133–900	386	204	659	18–45	30	24	38	40,446 ± 17,572
Acrylonitrile	218–1,177	541	303	830	15–50	27	21	37	49,480 ± 24,324
Nylon	130–5,540	5,180	770	5,400	15–42	25	19	32	369,331 ± 120,820

### Microplastic fiber surface roughness and wettability

**Fig 2:**
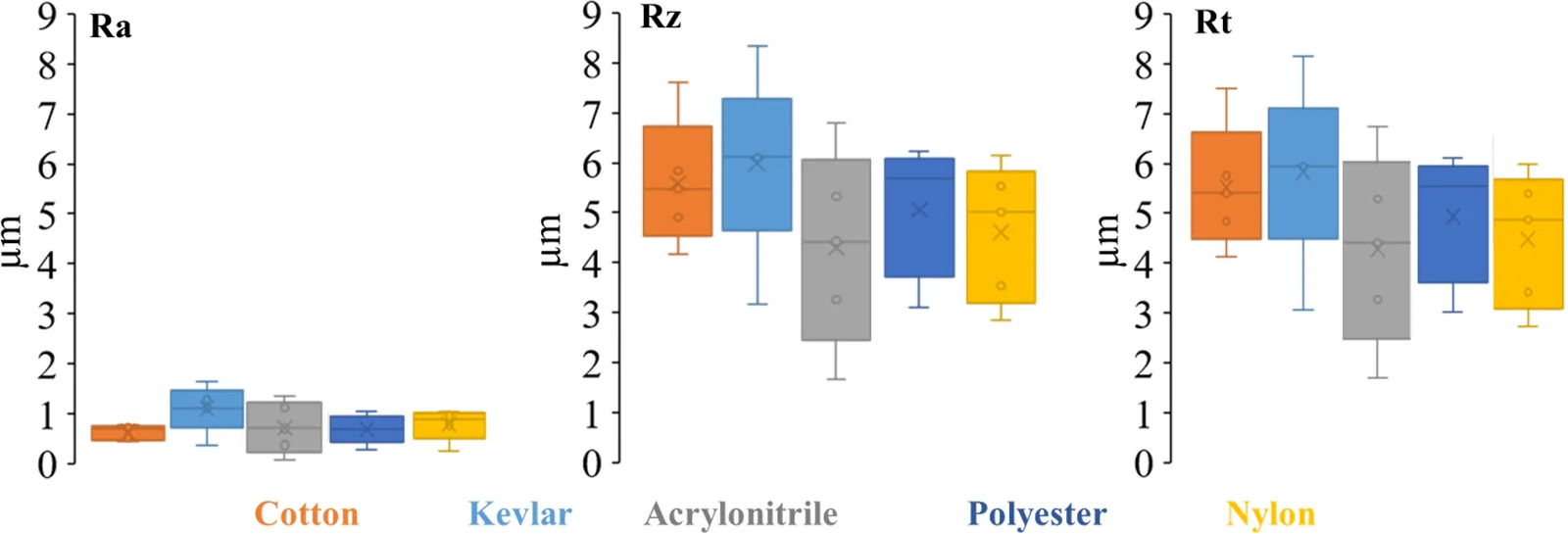
Box plots summarizing surface roughness parameters for the five pristine microplastic fibers. Ra = arithmetic mean height, Rz = maximum height, Rt = maximum profile height, box = interquartile range between first and third quartile (i.e., 50% of data), line = median, whiskers = minimum and maximum values.

Although there was no statistical difference within all materials, Kevlar exhibited the highest surface roughness, with an arithmetic Ra of 1.8 and Rz and Rt values of 7.5. Acrylonitrile displayed similar roughness values, with an Ra of 1.6 and Rz and Rt values of 6. Cotton, nylon, and polyester also had similar Ra values at 0.8, 1.1, and 1.2, respectively, and Rz and Rt values of 6.8, 6.5, and 6.0, respectively. Despite having relatively high Rz and Rt values, cotton fibers had the lowest Ra values, implying a relatively smooth surface. Overall, surface roughness tended to decrease in the following order: Kevlar > acrylonitrile > polyester > nylon > cotton (Fig. 2).

Wettability tests showed that cotton became hydrophilic after 1 min of contact with water, while polyester, acrylonitrile, and nylon exhibited strong hydrophilicity immediately. Kevlar was the only hydrophobic material. All synthetic fibers displayed low surface energy.

### Microbial colonization on microplastic fibers

**Fig 3:**
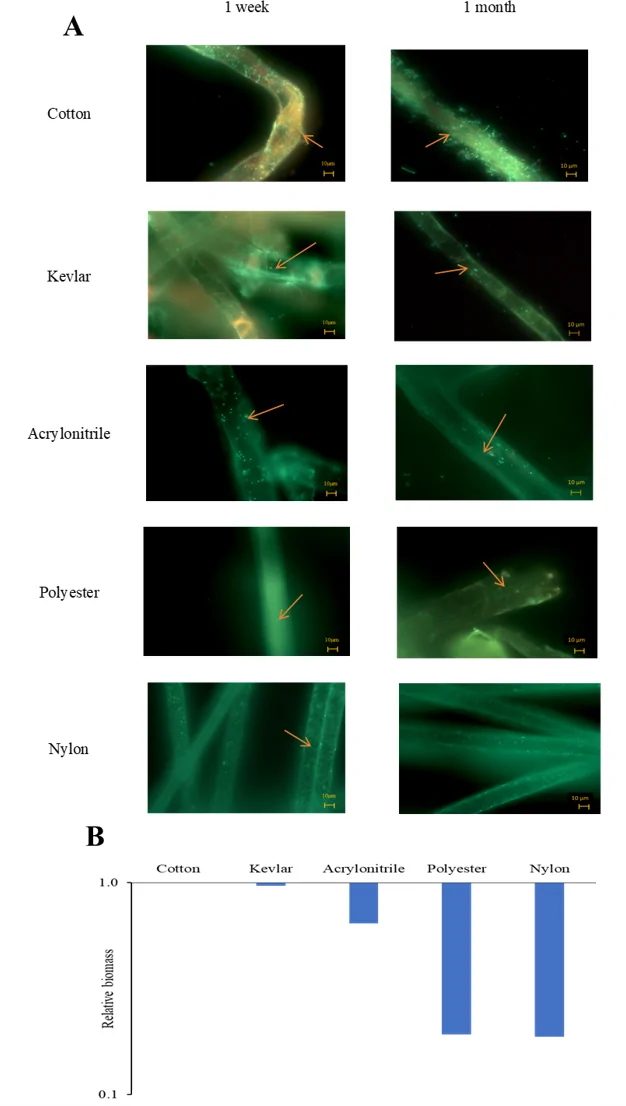
(**A**) Epifluorescence microscopy images of living bacteria (green dots) on microplastic fibers after 1 and 4 weeks of incubation. Scale bar = 10 µm. (**B**) Relative biomass determined by 16S rRNA after 1 month. Cotton acts as a reference sample with a relative abundance of 1. Note the logarithmic y-axis.

The epifluorescence microscopy confirmed adhesion of bacteria to all MPFs (Fig. 3A) after 1 week and 1 month. In general, more bacteria were observed on cotton, Kevlar, and acrylonitrile fibers than on polyester and nylon fibers. This was also confirmed by the relative biomass (Fig. 3B).

### Bacterial community composition

**Fig 4:**
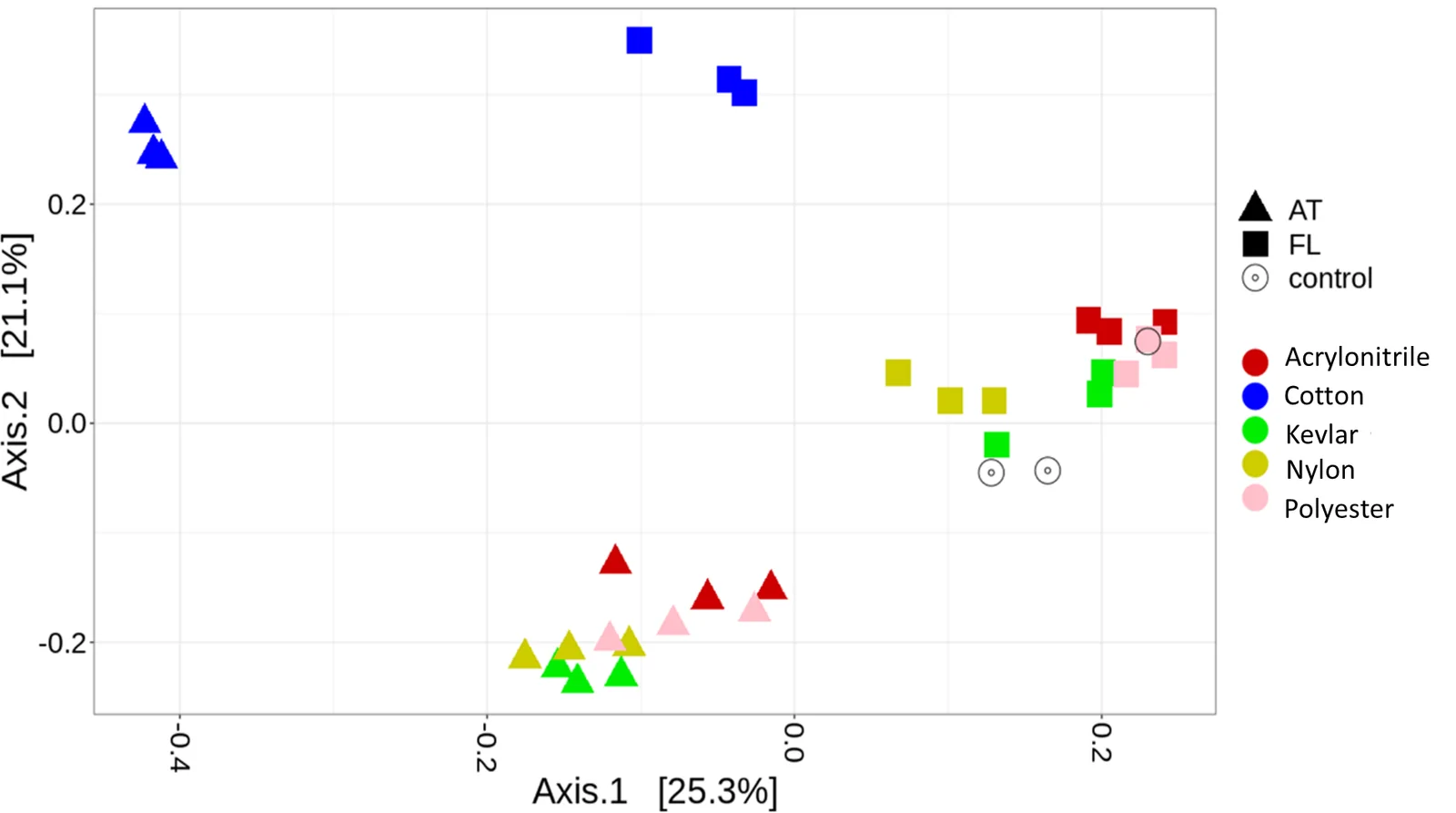
Principal coordinate analysis of Bray-Curtis dissimilarities between bacterial communities (genus level) on microplastic and cotton fibers after 1 month’s incubation (AT = attached bacteria, FL = free-living bacteria).

The PCoA revealed clear clustering of samples based on bacterial community composition (Fig. 4). First, attached bacteria and free-living bacteria for all MPFs formed distinct clusters with similar community compositions. Second, attached and free-living bacteria associated with cotton fibers displayed distinct profiles, clearly separating them from all polymer samples. Third, all attached microbial communities on MPFs differed significantly from the control. Finally, free-living bacteria in samples with MPFs were grouped closely with the control samples.

**Fig 5:**
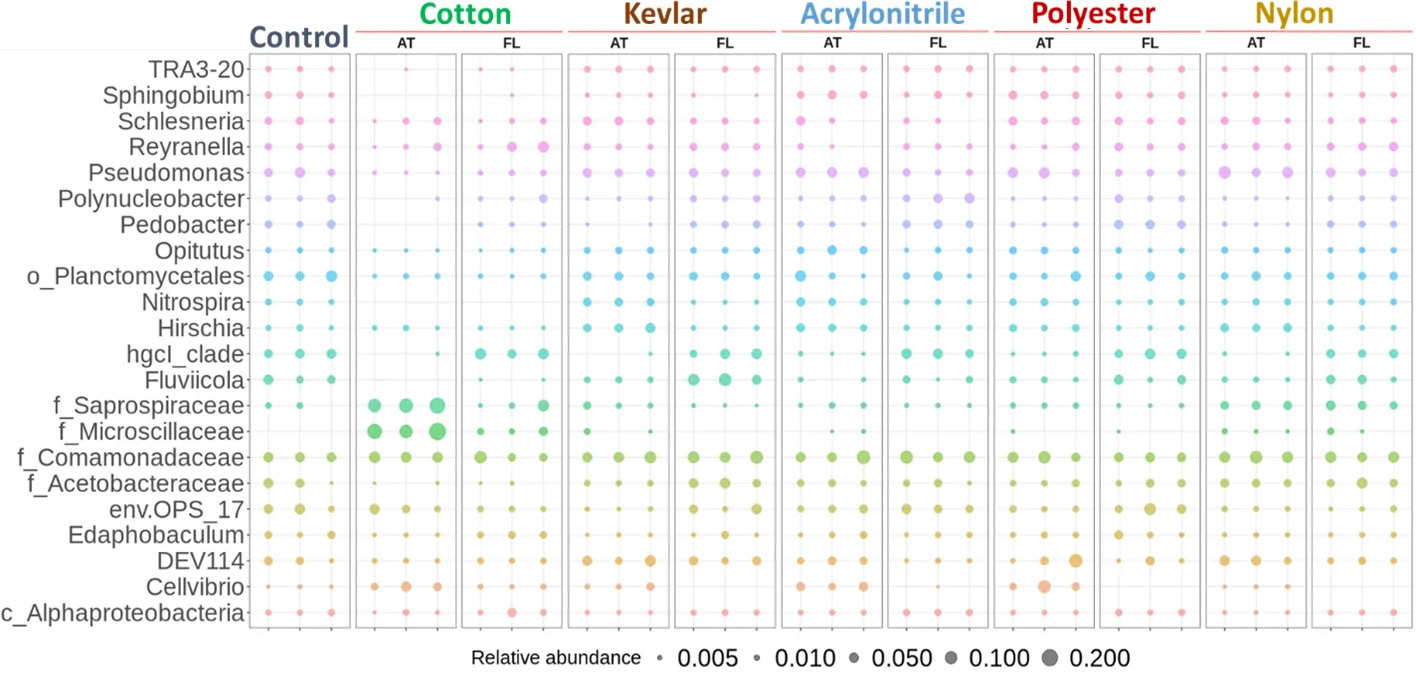
Bacterial communities (mean 0.01 genus level) on microplastic and cotton fibers after 1 month’s incubation (Control = solution without microplastic fibers, AT = attached bacteria, FL = free-living bacteria, circle size = proportional to relative abundance).

While some taxa were broadly distributed across all MPFs, others showed clear specificity toward certain materials (Fig. 5). For example, the family *Comamonadaceae* showed relatively high abundance across all MPFs, while the genus *Cellvibrio* and families *Saprospiraceae* and *Microscillaceae* were more abundant on cotton and nylon fibers than Kevlar, acrylonitrile, or polyester (Fig. 4). Both *Saprospiraceae* and *Microscillaceae* exhibited much higher abundances of attached bacteria than free-living bacteria in cotton samples, while *Microscillaceae* were almost absent from Kevlar, acrylonitrile, and polyester samples, particularly in the free-living fraction (Fig. 5). At the genus level, *Fluviicola* and *hgcl_clade* were dominant in free-living fractions across all MPFs, while others, such as *Hirschia* and *Nitrospira*, were more abundant in the attached fraction of synthetic MPFs. Genera such as *Opitutus*, *Pedobacter*, *Pseudomonas*, *Reyranella*, *Schlesneria*, *Sphingobium*, and TRA3-20 (*Burkholderiaceae*) exhibited variable abundance between attached and free-living fractions across MPFs (Fig. 5).

### Antibiotic resistance genes in microplastic fiber-attached bacteria

**Fig 6:**
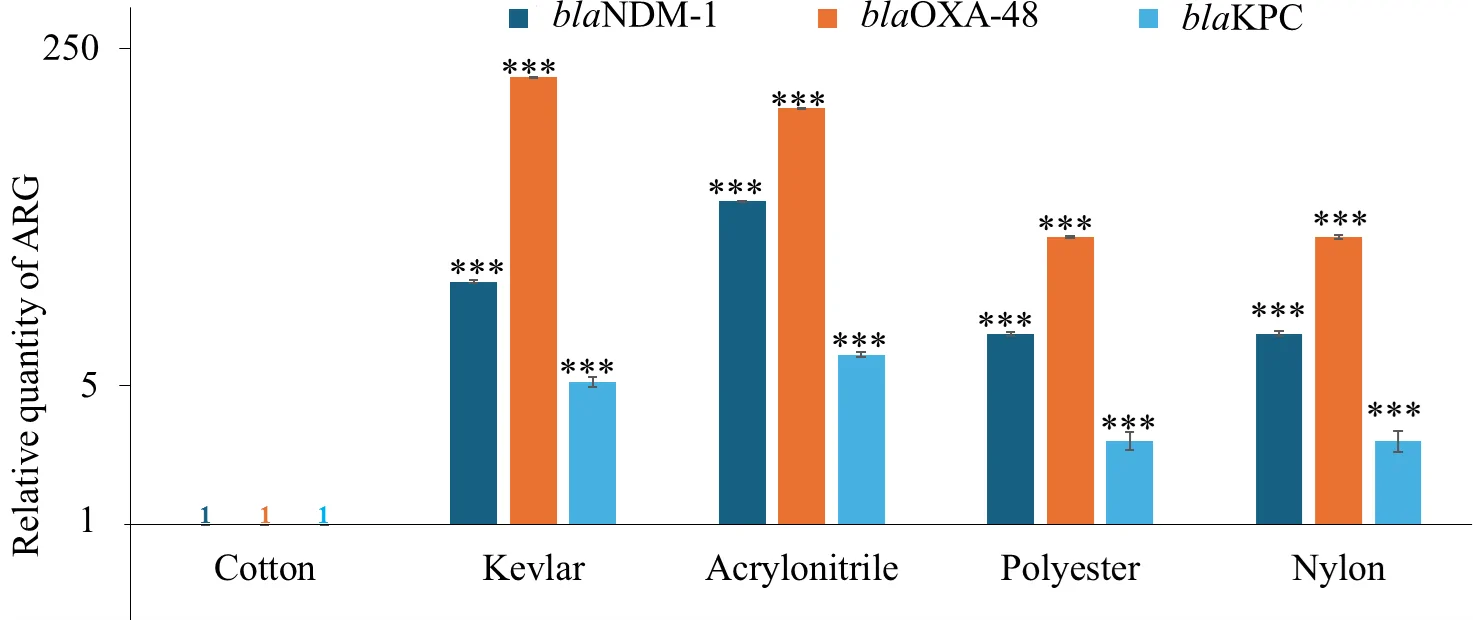
Relative abundance of antibiotic resistance genes (ARGs) in bacteria on microplastic (MPF) and cotton fibers. Cotton acts as a reference sample with a relative abundance of 1. Note the logarithmic y-axis. Significant differences (*P* < 0.05) between each ARG abundance in each fiber type compared to each ARG abundance in cotton, ***P < 0.0001.

When using the abundance of ARGs on cotton as a baseline to compare relative quantities with a value of 1 (Fig. 6), bacteria associated with MPFs showed a high prevalence of *bla*KPC, *bla*OXA-48, and *bla*NDM-1 ARG markers. The *bla*OXA-48 gene exhibited the greatest enrichment, with an abundance 180× higher on Kevlar, 120× higher on acrylonitrile, 60× higher on polyester, and 30× higher on nylon than on cotton (Fig. 6).

**Fig 7:**
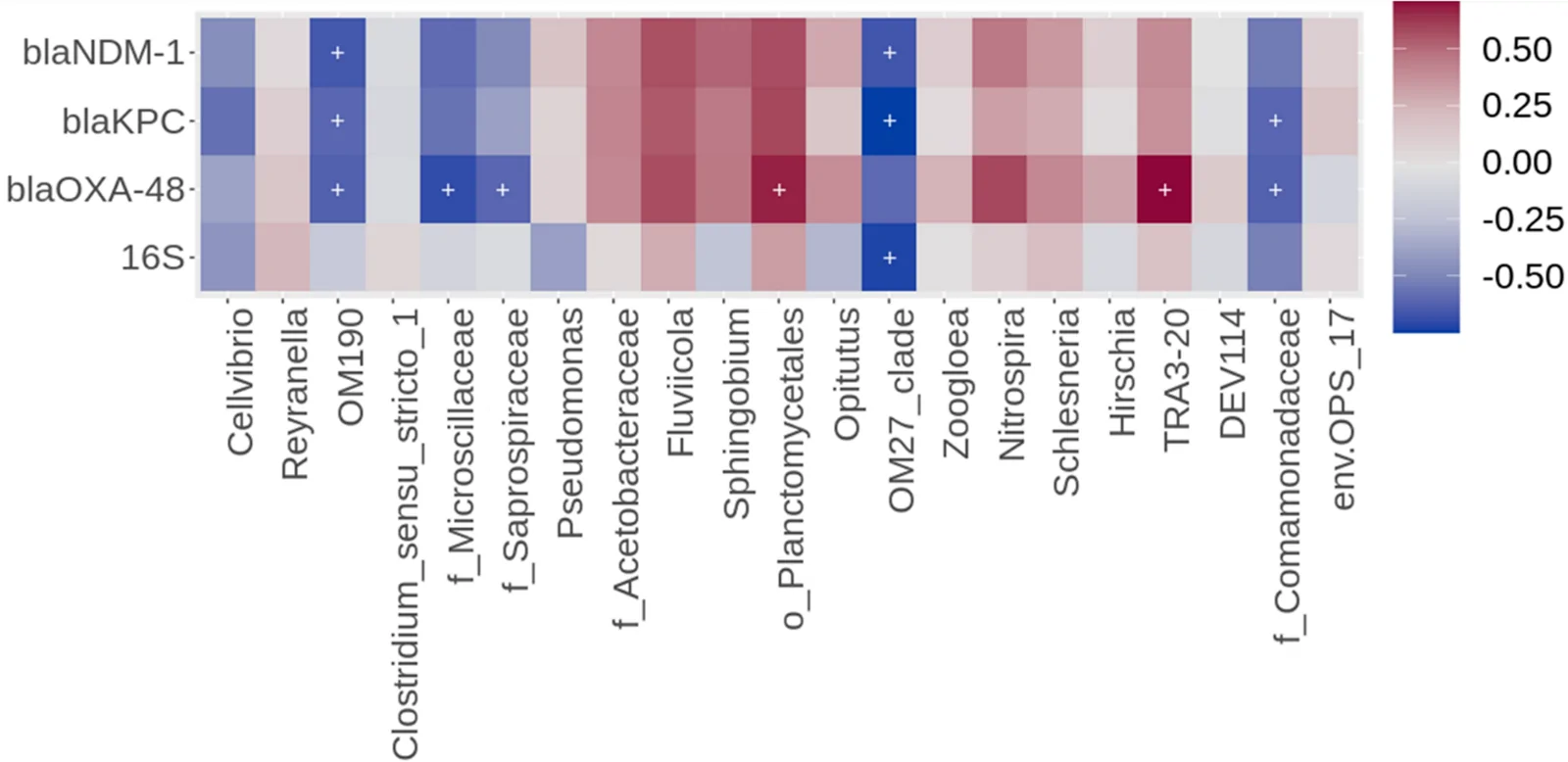
Correlations between antibiotic resistance gene relative abundance and biomass (16S) with the relative abundance of the dominant microbial taxa on both cotton and microplastic fibers. Blue = negative correlation, red = positive correlation, + = significant at *P*  <  0.05.

Bacteria attached to MPFs were found to have a higher positive correlation with ARGs than free-living bacteria (Fig. 7). Within the attached community, *Planctomycetales* and TRA3-20 (*Burkholderiaceae*) members showed a significant positive correlation with the *bla*OXA-48 gene (*P* < 0.05), followed by *Fluviicola* and *Nitrospira*. Both *bla*NDM-1 and *bla*KPC were predominantly associated with the abundance *of Fluviicola*, *Planctomycetales*, *Nitrospira*, *Schlesneria*, and TRA3-20 (Fig. 7). Interestingly, abundance of the *Comamonadaceae* family was negatively correlated with *bla*OXA-48 and with *bla*KPC in the MPF biofilm (*P* < 0.05).

## DISCUSSION

This study sheds new light on how natural and synthetic textile fibers influence microbial colonization and ARG enrichment in freshwater. While microplastics are known to serve as substrates for microbial attachment and facilitate horizontal gene transfer (42, 43), previous studies have primarily examined pellets or fragments rather than textile MPFs, which tend to dominate in microplastic emissions from laundering and wastewater effluent (44–46). This study compared the ARGs carried by bacterial communities attached to MPFs with those attached to natural cotton, thereby addressing a gap highlighted in plastisphere reviews (13, 47).

### Microplastic fiber-associated antibiotic resistance genes and their environmental implications

The increased abundance of all ARGs (*bla*NDM-1*, bla*OXA-48*,* and *bla*KPC) in bacteria attached to MPFs suggests (Fig. 6) that the presence of MPFs in water could indeed enhance the development and spread of antimicrobial resistance in aquatic ecosystems (25, 48). While previous studies have shown that ARGs are enriched on microplastics compared with natural surfaces, such as sand, wood, or rock (49, 50), a large meta-analysis by Yu et al. (51) revealed that ARGs were enriched in biofilms on natural organic substrates (e.g., leaves and wood) compared to microplastics. However, the same study also reported clear enrichment of human pathogenic bacteria carrying ARGs on the microplastics (51). These contradictory findings imply the need for more studies on ARG enrichment on different substrates, along with studies of ARG abundance, diversity, and host specificity.

Interestingly, our study revealed differences in ARG abundance between cotton and MPFs. Based on the parameters assessed, surface roughness does not appear to explain the observed differences, as no significant differences were detected among the materials (Fig. 1). Similarly, hydrophilicity is unlikely to be the sole driver because cotton is hydrophilic, as are the other MPFs except Kevlar. Surface area also did not show a clear relationship with ARG abundance. These observations suggest that material type may influence ARG enrichment, potentially through effects on early bacterial colonization and subsequent biofilm development. However, mechanistic insights into the processes responsible for ARG enrichment, including the potential role of horizontal gene transfer, were beyond the scope of this study. In this study, ARGs were analyzed in well-established, 1-month-old biofilms; therefore, we may presume that ARG abundance is likely driven by material type and the initial interactions with early colonizers. In addition, the bacterial communities in biofilms formed on MPFs and cotton differed. We observed a similar pattern with bio-based plastics, where the potential for horizontal transfer of ARGs was much higher among bacteria on plastics than on natural chitosan, although chitosan supported higher bacterial biomass (46).

Among the ARGs studied, the *bla*OXA-48 gene exhibited greatest enrichment (up to 180× compared to cotton) in bacteria on synthetic MPFs. This plasmid-associated ARG is of particular interest because it contains a carbapenemase-encoding gene that causes resistance to carbapenems (52). OXA-48-like enzymes, which were enriched on all MPFs in our study, have been confirmed in various bacterial taxa from environmental water worldwide, particularly in *Enterobacteriaceae*, *Pseudomonas*, and *Acetobacteraceae* (52).

The abundance of certain taxa within attached biofilms (e.g., *Planctomycetales,* TRA3-20) was positively correlated with the abundance of specific ARGs, suggesting possible selective enrichment and a higher potential for ARG spread within these bacteria. Notably, resistance to β-lactams has been detected in several strains within the *Planctomycetales*, including the ampicillin-resistant *Schlesneria* (53). These strains were also positively correlated with increased ARG abundance, although this correlation was not statistically significant.

### Microbial colonization and biofilm formation on MPFs

SEM analysis revealed clear differences in MPF morphology, with cotton, Kevlar, and acrylonitrile displaying frayed or roughened ends, whereas polyester and nylon had smoother ends. Fiber length and surface area differed significantly between materials, with nylon having the largest average surface area (369,331 µm²) and longest fibers, potentially offering a larger substrate for microbial colonization (42). However, after 1 month, nylon fibers harbored fewer bacteria than cotton, Kevlar, or acrylonitrile, despite their larger surface area. This suggests that surface roughness and chemical composition may have a stronger influence on initial microbial adhesion than surface area alone (54). In our study, the influence of surface roughness on microbial colonization was not confirmed, as it did not differ significantly among the tested materials.

Of the materials studied, cotton fibers exhibited the highest bacterial abundance (Fig. 3), suggesting that natural fibers promote greater microbial colonization than MPFs (55). Natural fibers are indeed more biodegradable by microorganisms because they can be metabolized to provide energy for bacterial growth (56). In the present study, cotton hosted a distinct microbial community with a higher representation of the bacterial families *Saprospiraceae* and *Microscillaceae. Saprospiraceae* have frequently been found in microplastic-contaminated environments in previous studies, consistent with their adaptability and widespread presence in both coastal and freshwater ecosystems (57, 58). *Microscillaceae* are also often found on microplastics, helping form “plastisphere” communities that may support nutrient cycling in aquatic ecosystems (59, 60). The dominance of these families on cotton fibers suggests that cotton may have physical properties that are well-suited to colonization by these bacteria.

In general, the alpha-diversity of bacteria colonizing MPFs did not differ greatly (Fig. 4), suggesting that material type had minimal influence. This finding is consistent with a study by Nguyen et al. (20), who found that the alpha-diversity of abundant taxa on microplastics submerged in a freshwater reservoir was not driven by microplastic material over a 2-month study period. The presence of families such as *Comamonadaceae, Microscillaceae,* and *Saprospiraceae* across multiple MPFs and cotton suggests that some taxa have broad ecological plasticity. However, TRA3-20*, Sphingobium, Polynucleobacter, Pedobacter, Nitrospira*, and *Fluviicola* were detected on all synthetic MPFs (Fig. 4), but not on cotton, or only rarely, suggesting that these genera may prefer synthetic materials to cotton. *Sphingobium,* in particular, has been reported as a potential microplastic degrader and appears capable of enhancing biofilm resilience under environmental stress, including the presence of antibiotics (61–63). The genus *Nitrospira,* an important bacterium in nitrogen cycling processes, has previously been observed on microplastics in freshwaters (64), as have *Fluviicola* (13, 20, 65), which showed enrichment on all MPFs, especially Kevlar (Fig. 4). Other notable colonizers, such as *Opitutus* and *Hirschia*, are known to form biofilms that support degradation processes (66, 67), while two other genera, *Pseudomonas* and *Pedobacter*, have shown strong potential for degrading plastics and may be involved in pathogenicity and antibiotic resistance, highlighting the need for further research (59, 68–72).

### Conclusion

This study revealed significant differences in microbial colonization and ARG enrichment on natural and synthetic fibers in freshwater environments. Although cotton fibers supported denser and more diverse biofilms, all MPFs (Kevlar, acrylonitrile, polyester, and nylon) showed far greater ARG enrichment, particularly for the *bla*OXA-48 gene, with abundance being up to 180× higher on Kevlar than on cotton. Despite lower bacterial colonization, synthetic MPFs acted as hotspots for ARG-harboring bacteria, including *Fluviicola, Sphingobium, Nitrospira*, and TRA3-20 (*Burkholderiaceae*). These findings indicate that ARGs were substantially more enriched on MPFs than on natural cotton fibers, emphasizing the elevated ecological risk posed by synthetic textiles, whose persistence and mobility may facilitate the long-term spread of antibiotic resistance in aquatic environments.

## Data Availability

Primary data from the sequencing of samples have been deposited in the NCBI under BioProject accession no. PRJNA1301084, available at https://www.ncbi.nlm.nih.gov/bioproject/PRJNA1301084.
